# Prognostic value of stress hyperglycemia ratio on short- and long-term mortality after acute myocardial infarction

**DOI:** 10.1007/s00592-022-01893-0

**Published:** 2022-05-09

**Authors:** T. Schmitz, D. Freuer, E. Harmel, M. Heier, A. Peters, J. Linseisen, C. Meisinger

**Affiliations:** 1 Chair of Epidemiology, University of Augsburg, University Hospital Augsburg, Stenglinstraße 2, 86156 Augsburg, Germany; 2 Department of Cardiology, Respiratory Medicine and Intensive Care, University Hospital Augsburg, Augsburg, Germany; 3 KORA Study Centre, University Hospital of Augsburg, Augsburg, Germany; 4 Institute for Epidemiology, Helmholtz Zentrum München, Ingolstädter Landstr. 1, 85764 Neuherberg, Germany; 5 Chair of Epidemiology, Institute for Medical Information Processing, Biometry and Epidemiology, Medical Faculty, Ludwig-Maximilians-Universität München, Munich, Germany; 6 German Center for Diabetes Research (DZD), Neuherberg, Germany; 7 Independent Research Group Clinical Epidemiology, Helmholtz Zentrum München, Ingolstädter Landstr. 1, 85764 Neuherberg, Germany

**Keywords:** Stress hyperglycemia, Admission glucose, Myocardial infarction, Short-term mortality, Long-term mortality

## Abstract

**Aims:**

Prior studies demonstrated an association between hospital admission blood glucose and mortality in acute myocardial infarction (AMI). Because stress hyperglycemia ratio (SHR) has been suggested as a more reliable marker of stress hyperglycemia this study investigated to what extent SHR in comparison with admission blood glucose is associated with short- and long-term mortality in diabetic and non-diabetic AMI patients.

**Methods:**

The analysis was based on 2,311 AMI patients aged 25–84 years from the population-based Myocardial Infarction Registry Augsburg (median follow-up time 6.5 years [IQR: 4.9–8.1]). The SHR was calculated as admission glucose (mg/dl)/(28.7 × HbA1c (%)—46.7). Using logistic and COX regression analyses the associations between SHR and admission glucose and mortality were investigated.

**Result:**

Higher admission glucose and higher SHR were significantly and nonlinearly associated with higher 28-day mortality in AMI patients with and without diabetes. In patients without diabetes, the AUC for SHR was significantly lower than for admission glucose (SHR: 0.6912 [95%CI 0.6317–0.7496], admission glucose: 0.716 [95%CI 0.6572–0.7736], *p*-value: 0.0351). In patients with diabetes the AUCs were similar for SHR and admission glucose. Increasing admission glucose and SHR were significantly nonlinearly associated with higher 5-year all-cause mortality in AMI patients with diabetes but not in non-diabetic patients. AUC values indicated a comparable prediction of 5-year mortality for both measures in diabetic and non-diabetic patients.

**Conclusions:**

Stress hyperglycemia in AMI patients plays a significant role mainly with regard to short-term prognosis, but barely so for long-term prognosis, underlining the assumption that it is a transient dynamic disorder that occurs to varying degrees during the acute event, thereby affecting prognosis.

**Supplementary Information:**

The online version contains supplementary material available at 10.1007/s00592-022-01893-0.

## Introduction

At time of acute myocardial infarction (AMI) hyperglycemia can occur due to increasing levels of catecholamines, steroids, glucagon and a decreasing level of insulin induced by stress, even in the absence of preexisting diabetes [1]. Previous studies reported a prevalence of stress hyperglycemia on admission in 20–50% of patients with ST- segment elevation myocardial infarction (STEMI) [2, 3]. Stress hyperglycemia has been defined as temporarily increasing plasma glucose levels in critically ill patients without a previously diagnosis of diabetes mellitus [4], but the exact definition of stress hyperglycemia in connection with an AMI has not been established [2, 3]. Hyperglycemia is a main determinant of atherosclerotic plaque instability and rupture and a trigger of hyperinflammation and endothelial dysfunction (inter alia via modulation of microRNA and apoptotic pathways [5]). Sardu et al. for instance, reported that stress hyperglycemia during STEMI may affect coronary thrombus composition, including increased inflammation such as elevated levels of tumor necrosis factor-α in coronary thrombi obtained from the hyperglycemic patients [6].

A number of studies have considered glucose concentrations on hospital admission (admission glucose) as a marker of stress hyperglycemia and have consistently demonstrated an association with both short- and long-term mortality in AMI patients with and without diabetes [2, 7–10], with some of the studies finding a stronger association in patients without diabetes [3, 11, 12]. In addition to mortality, hyperglycemia during the event appears to generally increase MACE (including re-hospitalization for coronary disease, heart failure, and stroke) [13].

However, admission glucose has been questioned as a reliable marker of stress hyperglycemia [7, 9], and therefore recently a new index, the stress hyperglycemia ratio (SHR), which is calculated from glucose and HbA1c at admission, was introduced [14].

So far, there are no studies simultaneously investigating and comparing the association of SHR and admission blood glucose with the short- and long-term mortality in AMI patients. In this study, based on data of the population-based Augsburg myocardial infarction registry, we examined the predictive effect of SHR and admission glucose on the 28-day mortality and long-term mortality (mainly 5-year mortality) in AMI patients with and without known diabetes.

## Methods

### Study population

This study was based on data from the Myocardial Infarction Registry that was established in Augsburg as part of the world health organization (WHO) project MONICA (Monitoring Trends and Determinants in Cardiovascular disease) in 1984. Since then all coronary deaths and cases of non-fatal AMI occurring among the inhabitants of the city of Augsburg and the two adjacent counties (more than 600,000 inhabitants) have been continuously registered. The population-based registry was included into the KORA (Cooperative Health Research in the Region of Augsburg) framework after termination of the MONICA project in 1995.

Patients aged between 25 and 84 years, being admitted to one out of eight hospitals in the study area were registered. Cases of hospitalized AMI that were recorded include: ST-elevation myocardial infarction (very regularly confirmed by a cardiac catheterization) and NSTEMI / NSTE-ACS events (unstable angina pectoris accompanied by a cardiac enzyme dynamic; in most cases also confirmed by a cardiac catheterization). More detailed information on case identification, diagnostic classification of events and quality control of the data can be found in previous publications [15, 16]. All study participants gave written informed consent. Both methods of data collection and questionnaires have been approved by the ethics committee of the Bavarian Medical Association (Bayerische Landesärztekammer) and the study was performed in accordance with the Declaration of Helsinki.

**Fig. 1:**
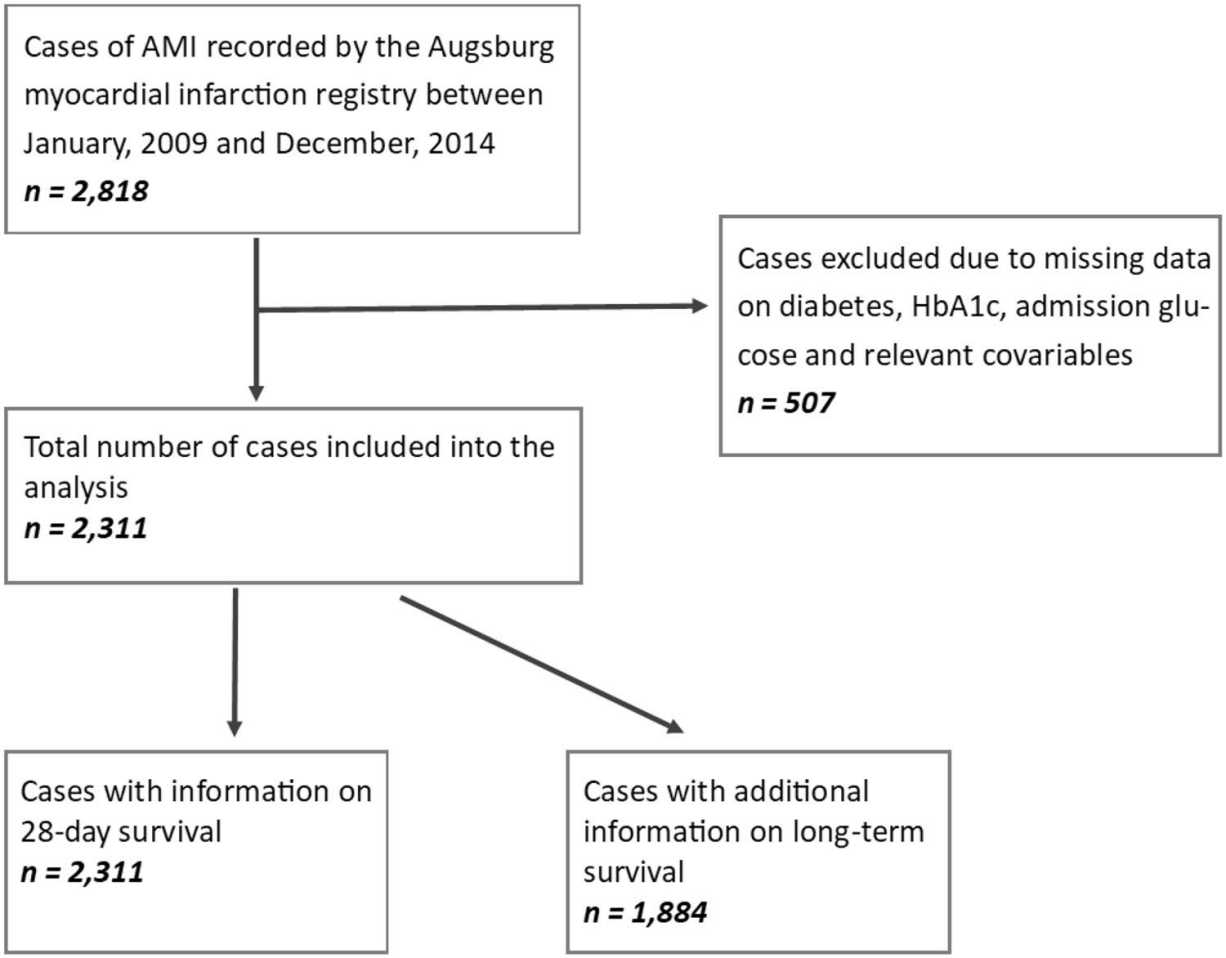
Flowchart displaying all inclusions and exclusions of cases

For the present study patients with AMI admitted to the university hospital of Augsburg between 2009 and 2014 were included, since blood from AMI patients was collected solely in this hospital. Participants with missing information on diabetes, HbA1c, admission glucose and relevant covariables (*n* = 507) were excluded. The final study population consisted of 2,311 patients with AMI. For all 2,311 patients information on 28-day survival was available. For a total of 1,884 patients who survived the first 28 days after the infarction, there was additional information on long-term survival. These patients were followed-up for a median time of 6.5 (IQR: 4.9–8.1) years. Figure 1 displays all inclusions and exclusions of cases for this study.

### Data collection

Trained study nurses interviewed the participants during hospital stay using a standardized questionnaire. In order to confirm the information provided by the patients and to collect additional information, the patients’ medical chart was reviewed. Demographic data, data on cardiovascular risk factors, medical history, comorbidities including diabetes, medication before and during hospital stay, as well as at discharge were collected from each patient. Furthermore, laboratory parameters including the glucose measurement upon admission or during hospital stay, ECG, and in-hospital course were determined as well.

During the interview, all patients were asked whether they have a diabetes mellitus disease. The presence of diabetes mellitus in a patient was, furthermore, extracted from the medical chart. The patient was assigned to the non-diabetes group when there was no indication for an existing diabetes mellitus neither in the interview nor in the medical chart. All other cases were assigned to the diabetes group. HbA1C values were not used for diabetes grouping.

For any in-hospital complication including cardiogenic shock, left ventricular decompensation, bradycardia, in-hospital reinfarction, ventricular tachycardia, and ventricular fibrillation, one variable was generated (yes/no).

One further variable was generated whether the patient received all four evidence-based medications (EBM) at discharge (antiplatelet drug, ACE blockers/ATII antagonist, beta-blockers, statins).

Between 2009 and 2014 plasma samples were collected from AMI cases during hospital stay and frozen at −80 °C.

### Clinical chemistry measurement

HbA1c values were measured in stored samples with a reverse-phase cation-exchange high-pressure liquid chromatography (HPLC) method (Analyzer HA 8160; Menarini, Florence, Italy). All other blood parameters were measured in all AMI patients during hospital stay as part of the treatment.

### Outcome

The endpoints used in this study were case-fatality within 28 days and long-term all-cause mortality. Mortality was ascertained by regularly checking the vital status of all registered persons of the MI registry through the population registries. Death certificates were obtained from local health departments.

### Statistical analysis

For categorical variables, Chi-square test or Fisher’s exact test were performed and the results were presented as absolute frequencies with percentages. For normally distributed continuous variables, Student’s t-tests were used and the results were presented as means with standard deviations. For continuous variables that were not normally distributed, nonparametric tests were used and the results were presented as medians with inter-quartiles ranges.

For all analyses concerning long-term survival, only patients who survived the first 28 days after the infarction were included in order to solely concentrate on long-term mortality.

### ROC

To assess the accuracy of the prediction of short- and long-term mortality, ROC curves (receiver operating characteristic) and AUC (area under the curve) values were calculated. As this requires dichotomous outcome variables, we choose 28-day survival for short-term and 5-year survival for long-term mortality as the outcome variables. For the 5-year ROC, all cases with missing information on 5-year survival were removed from the analysis. These cases were included back in for the subsequent survival time analyses applying Cox proportional-hazards regression. To compare the discriminatory power between SHR and admission glucose, bootstrapping methods were used to calculate p-values (number of bootstrap replicates: 2,000).

### Logistic and Cox regression models

To further examine the association between SHR/admission glucose and short- and long-term mortality, multivariable logistic regression and Cox regression models were calculated. According to a literature review the models were adjusted for the following confounders: age, sex, typical chest pain symptoms, smoking, hyperlipidemia, hypertension, impaired renal function (according to GFR), PCI, bypass surgery, and lysis therapy. All regression models included an interaction-term between SHR/admission glucose and the dichotomous variable known diabetes.

To capture a possible nonlinear relationship the variables SHR and admission glucose were modeled as restricted cubic splines (RCS) with 4 knots (at the 5th, 35th, 65th, and 95th percentiles).

All statistical analyses were carried out with the R program version 4.1.0. A p value of < 0.05 was considered as statistically significant.

## Results

### Demographic characteristics

Baseline characteristics of AMI patients with and without diabetes are summarized in Table 1. During 28 days after hospital admission, 154 patients (50 with and 104 without known diabetes) had died. Among the AMI patients surviving the first 28 days after the event, 188 patients with and 245 patients without known diabetes had died during follow-up. At baseline, the mean age of AMI patients with diabetes was significantly higher than in non-diabetic patients (68.0 vs. 63.7 years). Patients with diabetes more often had hypertension and hyperlipidemia but were less often current smokers than patients without diabetes. Furthermore, patients with diabetes more often presented with a NSTEMI; they more often had an impaired kidney function, decreased admission hemoglobin, and higher levels of admission C-reactive protein values compared to patients without known diabetes.

**Table 1:** Baseline characteristics of patients with and without diabetes. Categorical data presented as total numbers (%). Numeric data is presented as mean (SD) or median (IQR)

	Diabetes *n* = 681 (29.5%)	No Diabetes *n* = 1630 (70.5%)	*p*-value	*n*
28-day case fatality (%)	50 (7.3)	104 (6.4)	0.451	2311
Total number of cases with information on long-term survival (row percentage)	561 (29.8)	1323 (70.2)		1884
Number of deaths between 28 days and 5 years (%)	127 (25.7)	133 (11.5)	< 0.001	1650
Number of deaths after 28 days (%)	188 (33.5)	245(18.5)	< 0.001	1884
Sex (male)	481 (70.6)	1203 (73.8)	0.1305	2311
Age (mean, SD)	68 (11)	63.7 (12.7)	< 0.001	2311
Admission Glucose (mg/dl) (median, IQR)	178 (142–237)	125 (108–150)	< 0.001	2311
HbA1c (%) (median, IQR)	6.6 (6–7.5)	5.6 (5.4–5.9)	< 0.001	2311
HbA1c (mmol/mol) (median, IQR)	48.6 (42.0–58.5)	37.7 (35.5–41.0)	< 0.001	2311
SHR (median, IQR)	1.2 (1.0–1.5)	1.1 (1.0–1.3)	< 0.001	2311
*Comorbidities and risk factors*				
Hypertension	611 (89.7)	1168 (71.7)	< 0.001	2311
Hyperlipidemia	416 (61.1)	796 (48.8)	< 0.001	2311
*Smoking status*				
- Current smoker	170 (25)	590 (36.2)	< 0.001	2311
- Never smoker	210 (30.8)	431 (26.4)	–	–
- Ex smoker	232 (34.1)	452 (27.7)	–	–
- No information on smoking	69 (10.1)	157 (9.6)	–	–
*Clinical characteristics*				
Typical chest pain symptoms	570 (83.7)	1403 (86.1)	0.2415	2311
Prehospital time in minutes (median, IQR)	139.5 (76.8–350)	122.5 (79–297.5)	0.0867	1996
*Type of infarction*				
- STEMI	355 (52.1)	970 (59.5)	0.0028	2311
- NSTEMI	260 (38.2)	556 (34.1)	–	–
- BBB	51 (7.5)	78 (4.8)	–	–
- No information on infarction type	15 (2.2)	26 (1.6)	–	–
*Renal function*				
- GFR > = 60 ml/min	382 (56.1)	1150 (70.6)	< 0.001	2311
- GFR 30-59 ml/min	246 (36.1)	425 (26.1)	–	–
- GFR < 30 ml/min	53 (7.8)	55 (3.4)	–	–
*LVEF*				
- > 50%	295 (43.3)	798 (49)	0.0498	2311
- 41–50%	140 (20.6)	319 (19.6)	–	–
- 31–40%	150 (22)	302 (18.5)	–	–
- ≤ 30%	65 (9.5)	123 (7.5)	–	–
- No information	31 (4.6)	88 (5.4)	–	–
Any in-hospital complication	172 (25.3)	395 (24.2)	0.6395	2311
Days at intensive care (mean, SD)	3.2 (5.2)	2.9 (4.6)	0.1705	2293
Days at intensive care (median, IQR)	2 (1–3)	2 (1–3)	0.1705	2293
*Laboratory values*				
Admission hemoglobin (g/l) (median, IQR)	139 (126–150)	143 (132–152)	< 0.001	2311
Admission troponin I (ng/ml) (median, IQR)	0.680 (0.13–6.35)	0.605 (0.11–4.6575)	0.8477	1951
Admission CRP (mg/dl) (median, IQR)	0.55 (0.29–1.45)	0.34 (0.29–0.85)	0.0507	2311
*Therapy*				
PCI	571 (83.8)	1401 (86)	0.2426	2311
Bypass therapy	90 (13.2)	188 (11.5)	0.4261	2311
Lysis therapy	4 (0.6)	15 (0.9)	0.702	2311
Any revascularization therapy	639 (93.8)	1539 (94.4)	0.6512	2311
All four evidence-based medications at discharge	510 (74.9)	1260 (77.3)	0.2325	2311

Median admission glucose levels were significantly higher in patients with diabetes (178 mg/dl) in comparison with non-diabetic patients (125 mg/dl). Also, median HbA1c and SHR values were higher in patients with versus patients without diabetes (HbA1c: 6.6% (48.6 mmol/mol) vs. 5.6% (37.6 mmol/mol); SHR: 1.21 vs. 1.11). In the supplementary material we additionally provide detailed information on diabetes medication before the event and anti-ischemic and diabetes medication at discharge (see supplementary material, Table 1).

### Short-term mortality

**Fig. 2:**
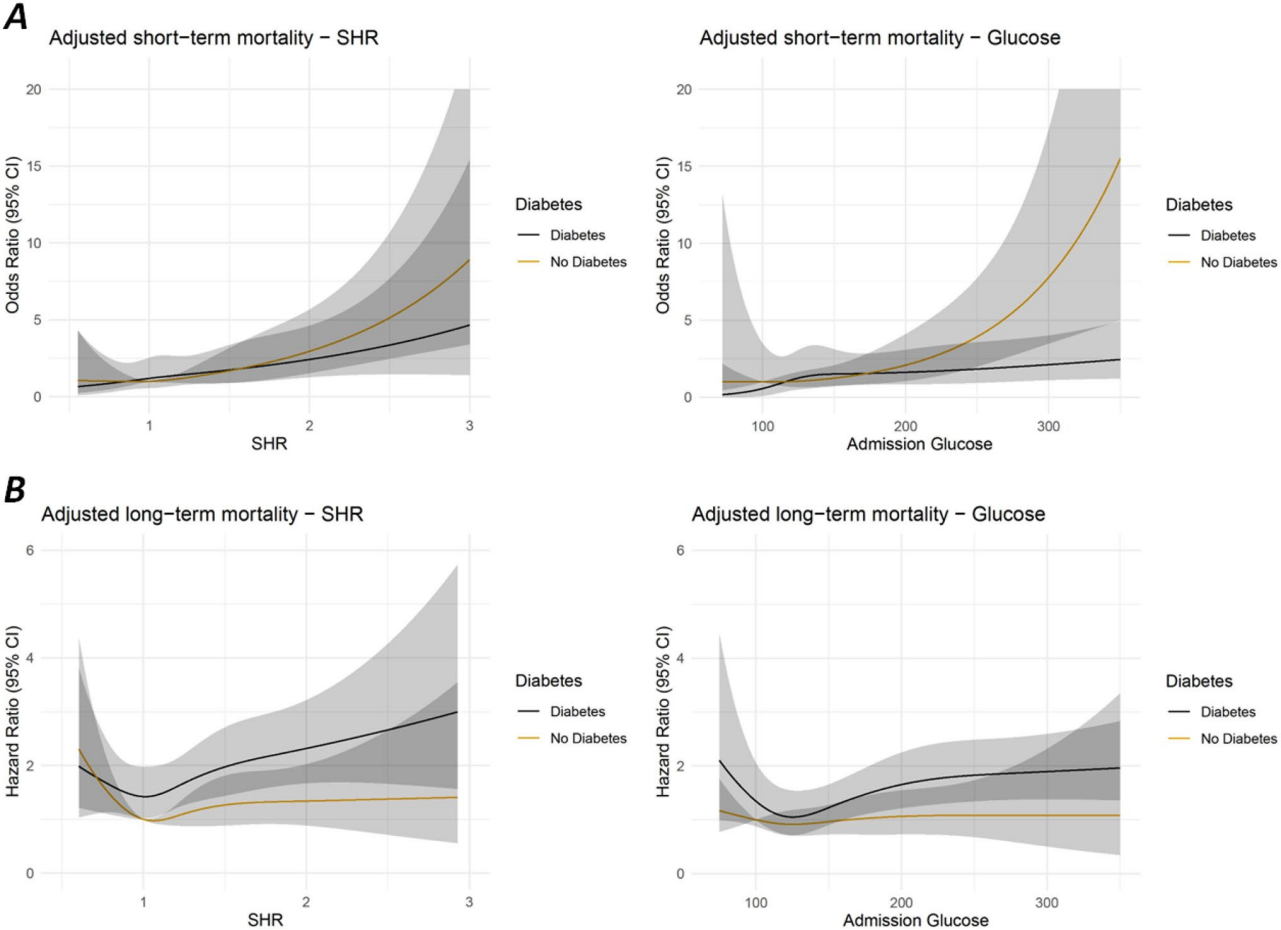
Nonlinear associations between SHR and admission glucose levels and short-term mortality **A** as well as long-term mortality **B**, respectively. All models include an interaction term with diabetes and were adjusted for age, sex, typical chest pain symptoms, smoking, hyperlipidemia, hypertension, impaired renal function (according to GFR), PCI, bypass surgery, lysis therapy

The association between adjusted SHR as well as admission glucose and 28-day mortality was modeled by using restricted cubic splines to show their dose–response relationship. As illustrated in Fig. 2A, SHR and admission glucose were nonlinearly associated with short-term mortality in the multivariable adjusted models in patients with and without diabetes. However, the associations were more pronounced in non-diabetic patients (see Figs. 2A and 4A).

**Fig. 3:**
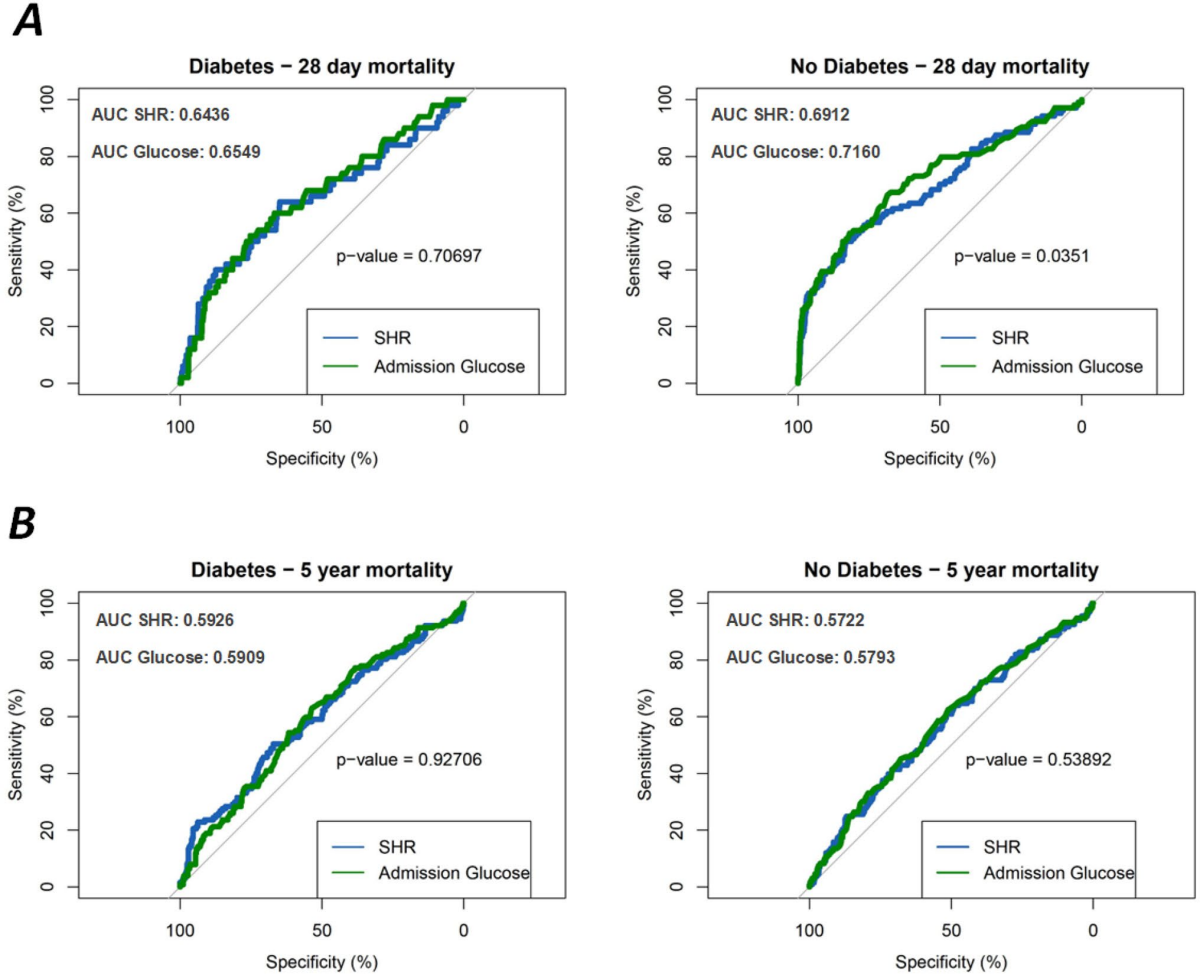
**A** ROC curves for prediction of 28 day mortality (diabetes patients in the left, non-diabetes patients on the right). **B** ROC curves for prediction of 5-year mortality (diabetes patients in the left, non-diabetes patients on the right). *P*-values for the comparison of SHR and admission glucose are calculated by comparing the AUC values using bootstrapping

In Fig. 3A the ROC curves for 28-day mortality are shown. For the patients with diabetes the AUC was 0.6436 [95%CI 0.5563–0.7309] for SHR and 0.6549 [95%CI 0.5721–0.7336] for admission glucose yielding no significant difference (p-value: 0.7070). For patients without diabetes, admission glucose predicted the short-term mortality significant better than SHR (AUC SHR: 0.6912 [95%CI 0.6317–0.7496], AUC admission Glucose: 0.716 [95%CI 0.6572–0.7736], *p*-value: 0.0351).

### Long-term mortality

**Fig. 4:**
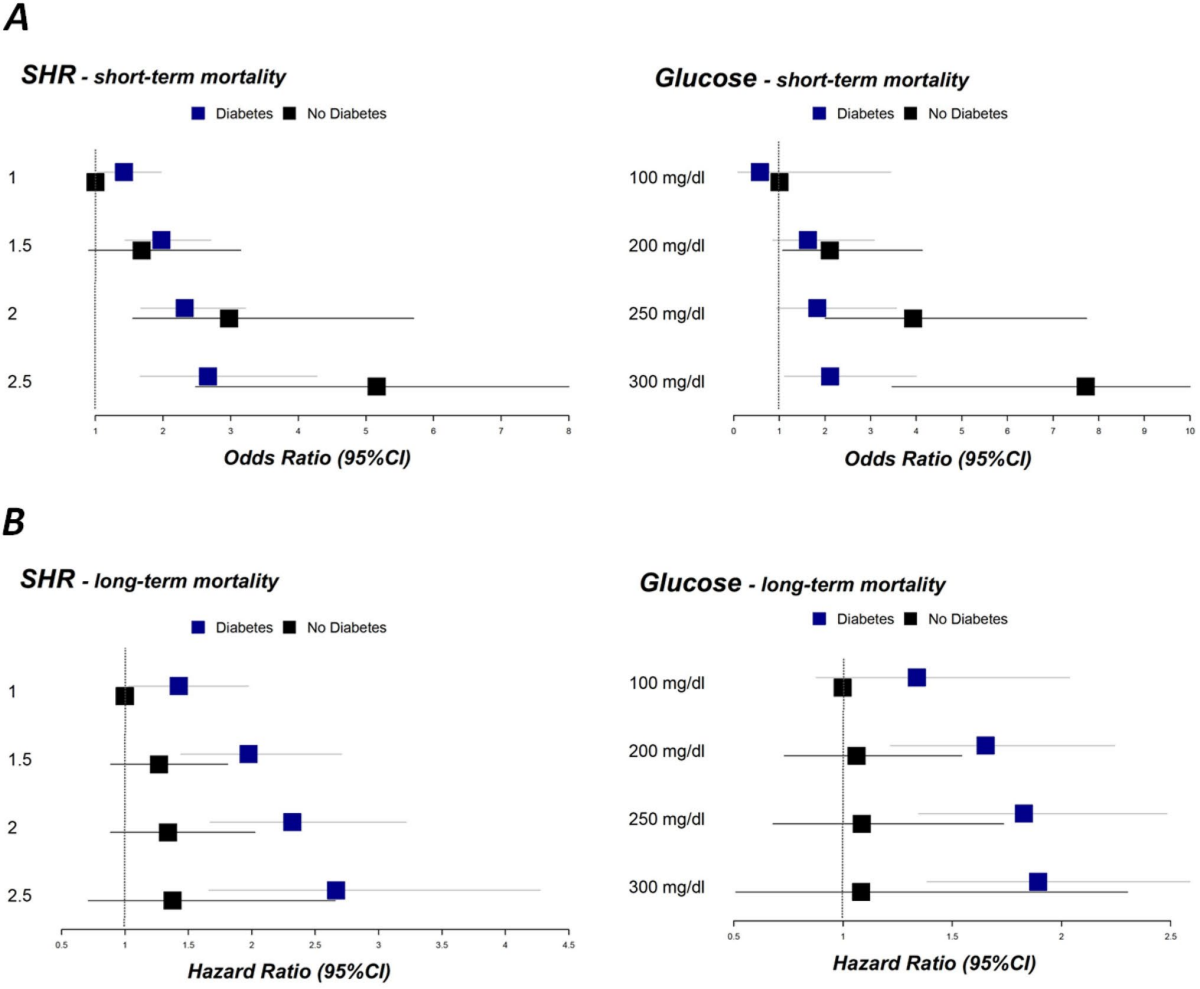
**A** Forest plots display several odds ratio values of the adjusted logistic regression model for selected values for SHR and admission glucose (short-term mortality). **B** Display of Hazard ratio values of the adjusted COX regression models for selected values for SHR and admission glucose (long-term mortality)

Figure 2B shows the nonlinear associations between SHR and admission glucose and the outcome in multivariable adjusted COX regression models in patients with and without diabetes. Overall, mortality was higher in patients with diabetes compared to those without diabetes in all models. Increasing admission glucose and increasing SHR were significantly associated with a higher 5-year all-cause mortality in AMI patients with diabetes but not in non-diabetic patients (Fig. 4B).

Figure 3B displays the ROC curves for 5-year mortality. The AUC for the patients with diabetes was 0.5926 [95%CI 0.5334–0.6495] for SHR and 0.5909 [95%CI 0.5329–0.6484] for admission glucose (*p*-value: 0.921). For the patients without diabetes, the AUC values were 0.5722 [95%CI 0.5178–0.6257] for SHR and 0.5793 [95%CI 0.5269–0.6313] for admission glucose (*p*-value: 0.5389). Overall, the AUC values indicated a comparable prediction of 5-year mortality for both measures in patients with and without diabetes with no significant differences.

For both, short-term and long-term mortality we performed a subgroup analysis with three diabetes groups instead of two. The diabetes group included all patients with known diabetes or HbA1C values ≥ 6,5%; the no diabetes group included patient without known diabetes and HbA1C values < 5.7%. The third group (prediabetes group) consisted of all patient without known diabetes but HbA1C values between 5.7 and 6.4%. In the supplementary material we display data on main characteristics and mortality as well as ROC curves stratified for the three diabetes groups (see supplementary material, table 2 and Fig. 1). It shows that short- and long-term mortality in the prediabetes group was higher than in the no diabetes group.

## Discussion

The present study found that SHR and glucose on admission were significantly associated with higher short-term mortality in both, diabetic and non-diabetic AMI patients. An association with higher long-term mortality was only seen in patients with diabetes. The predictive ability of SHR was roughly comparable to the predictive effect of admission glucose on both outcomes in both groups of patients. It appeared that stress hyperglycemia plays a significant role mainly regarding short-term prognosis, in particular, in non-diabetic patients, but not for long-term prognosis. To our knowledge, this study is the first one to investigate the significance of SHR in comparison with admission glucose on short- and long-term mortality in AMI patients with and without known diabetes mellitus.

A number of previous studies found an increased mortality and morbidity in AMI patients with hyperglycemia at admission, regardless whether they had diabetes or not [2, 9, 17–22]. However, some of the previous studies found that hyperglycemia in acute AMI was more likely to be associated with worse outcomes in non-diabetic patients than in patients with known diabetes [3, 11, 12].

There is controversy about whether elevated blood glucose on admission is merely an expression of severe disease or whether hyperglycemia is causally associated with serious consequences such as complications or death [23–25]. There are several eligible pathophysiological mechanisms potentially mediating a causal relationship. On the one hand, hyperglycemia during the AMI is supposed to be a main determinant of atherosclerotic plaque instability and rupture [5] and furthermore affects coronary thrombus composition [6] and increase coronary thrombus burden via thrombus microbiota dysbiosis [26]. On the other hand, hyperglycemia might exhibit sympathetic over-activity with a hyperglycemia-mediated proinflammatory pathway and in this affecting outcome [27].

Some prior studies assumed that hyperglycemia on admission was due to previously undiagnosed diabetes in myocardial infarction patients. However, this assumption was not confirmed in an investigation by Ishihara et al. [28]. Because admission glucose was questioned as a marker for stress hyperglycemia, SHR, an index of relative glycemia was introduced for it [14] with the aim of gaining new insights into the relationship between hyperglycemia and patient group outcomes by correcting glucose levels for HbA1c [29]. However, whether SHR is a better predictor of short- and long-term mortality in non-diabetic and diabetic AMI patients compared with (absolute) levels of admission glucose is not clear to date. In a recently published study by Chen et al. that included 345 elderly patients (> 75 years) with AMI, SHR was found to be nonlinearly related to in-hospital complications and in-hospital mortality. SHR was significantly associated with adverse outcome only in AMI patients without known diabetes, but not in patients with diabetes [30]. Another study including 4,362 Korean patients who underwent percutaneous coronary intervention (PCI) reported that SHR was a useful marker to predict MACCE after PCI particularly in patients with STEMI [31]. In the only study to date that examined the association between SHR and long-term prognosis in AMI patients, SHR was associated with a worse outcome (all-cause mortality and admission due to heart failure) in non-diabetic patients with STEMI. There was no significant relationship with the outcome in patients without diabetes [32].

In accordance with these studies, in our study, SHR was significantly associated with short-term mortality in non-diabetic and diabetic patients with AMI, but the relationship was more pronounced in non-diabetic patients. Regarding the long-term all-cause mortality in AMI patients SHR was significantly associated with the outcome in patients with known diabetes only. However, our study showed that SHR as a measure of relative hyperglycemia was not superior to admission glucose in predicting short- and long-term prognosis—in both diabetic and non-diabetic patients. Interestingly, the proportion of AMI patients who died within 28 days did not differ significantly between patients with and without diabetes in the present investigation. However, the mortality of AMI patients with known diabetes was significantly higher than that of nondiabetic patients in terms of long-term mortality (25.7% versus 11.5% of the patients died in the time period between 28 days and 5 years after AMI) so that the influence of an increased SHR or a high admission glucose at the time of the acute event on the long-term prognosis may be rather small. One plausible explanation may be that stress hyperglycemia is, by definition, a transient, often dynamic disorder that responds to changes in disease progression during the acute event. In contrast, other factors, such as severe comorbidities, which are particularly prevalent in diabetic patients, might play a more important role in long-term prognosis. One further point to consider is that the extent of insulin resistance might substantially affect long-term outcome after AMI even independent from elevated glucose and HbA1C levels. A study by Sasso et al. indicated that insulin resistance is related to restenosis and new PCI even in patients with normal glucose tolerance [33]. This effect is neither captured by SHR nor by glucose levels alone and therefore lowering predictive value of both markers with regards to long-term mortality.

### Strengths and limitations

The strengths of the present large study include that consecutive patients were recruited prospectively and followed up for a median time of 6.5 (IQR: 4.9–8.1) years. Standardized data collection was performed by conducting patient interviews during hospital stay and patient chart review. However, this study also has limitations. The first concerns the grouping of the diabetes group, as the no diabetes group also includes patients with HbA1C values ≥ 5.7%, which might have influenced the results. We addressed this problem by a subgroup analysis (see supplementary material, table 2 and Fig. 1). Because this is an observational study, causality could not be proven. It is further possible that some confounders, which could have influenced the associations were not available and could therefore not be included in the analysis. Thus, residual confounding cannot be entirely excluded. Since only patients between 25 and 84 years were included, results cannot be applied to older age-groups. Furthermore, the present findings may not be generalized to all ethnic groups.

### Conclusions

In conclusion, stress hyperglycemia seems to play a significant role mainly with regard to short-term mortality, but barely for long-term mortality in AMI patients, underlining the assumption that it is a transient dynamic disorder that occurs to varying degrees during the acute event, thereby affecting prognosis. In general, SHR does not appear to be a better predictor of prognosis in AMI patients compared to admission glucose. Especially in non-diabetic AMI patients, admission glucose is a reliable parameter for the assessment of stress induced hyperglycemia with proper predictive ability for short-term mortality. As it can be determined easier and more quickly than SHR, it remains the preferable parameter for early risk stratification in AMI patients compared to the novel SHR.

## Supplementary Information

Below is the link to the electronic supplementary material.

Supplementary file1 (DOCX 320 KB)

## Data Availability

The datasets generated during and/or analyzed during the current study are not publicly available due to data protection aspects but are available in an anonymized form from the corresponding author on reasonable request.
