# Supplementary material for: Prognostic value of stress hyperglycemia ratio on short- and long-term mortality after acute myocardial infarction
Source: Acta Diabetol. 2022 May 9;59(8):1019–29. doi: 10.1007/s00592-022-01893-0 (PMC9242951; doi:10.1007/s00592-022-01893-0)
Supplement: Supplementary file 1 — Supplementary file1 (DOCX 320 KB) [file 592_2022_1893_MOESM1_ESM.docx]

***Supplementary Material***

**Title:** Prognostic value of stress hyperglycemia ratio on short- and long-term mortality after acute myocardial infarction

***Table 1:*** *Medication use of before the event and at discharge for patients with diabetes and without diabetes*

|  | ***Diabetes***  n= 681 (29.5%) | ***No Diabetes***  n = 1630 (70.5%) | ***p-value*** | ***n*** |
| --- | --- | --- | --- | --- |
| ***Medication before the event*** | | | | |
| Statin | 122 (32.5) | 146 (18) | < 0.001 | 1187 |
| oral antidiabetics | 180 (47.9) | 3 (0.4) | < 0.001 | 1188 |
| GLP-1 receptor agonists | 4 (1.1) | 0 (0) | < 0.001 | 1187 |
| insulin | 79 (21) | 2 (0.2) | < 0.001 | 1190 |
| ***Medication at discharge*** | | | | |
| antiplatelet drug | 607 (99.3) | 1483 (99.2) | 0.9374 | 2106 |
| ACE blockers/ ATII antagonist | 559 (91.5) | 1352 (90.4) | 0.4998 | 2106 |
| beta-blockers | 582 (95.3) | 1440 (96.3) | 0.3109 | 2106 |
| Statin | 575 (94.1) | 1430 (95.7) | 0.1637 | 2106 |
| oral antidiabetics | 296 (48.4) | 7 (0.5) | < 0.001 | 2106 |
| GLP-1 receptor agonists | 9 (1.5) | 0 (0) | < 0.001 | 2106 |
| insulin | 163 (26.7) | 8 (0.5) | < 0.001 | 2106 |

***Table 2:*** Main characteristics and mortality according to three diabetes groups: ‘known diabetes’, ‘no diabetes - HbA1C ≥5.7%’ and ‘no Diabetes - HbA1C <5.7%’

|  | ***Diabetes***  n=770 (33.3%) | ***Prediabetes***  n=620 (26.8%) | ***No Diabetes***  n=921 (39.9%) | ***p-value*** | ***n*** |
| --- | --- | --- | --- | --- | --- |
| 28-day case fatality (%) | 62 (8.1) | 45 (7.3) | 47 (5.1) | 0.04192 | 2311 |
| total number of cases with information on long-term survival (row percentage) | 620 (32.9) | 498 (26.4) | 766 (40.7) |  | 1884 |
| number of deaths between 28 days and 5 years (%) | 135 (24.6) | 55 (12.6) | 70 (10.5) | < 0.001 | 1650 |
| number of deaths after 28 days (%) | 205 (33.1) | 106 (21.3) | 122 (15.9) | < 0.001 | 1884 |
| sex (male) | 545 (70.8) | 439 (70.8) | 700 (76) | 0.0222 | 2311 |
| age (mean, SD) | 68 (11) | 65.2 (12.4) | 62.4 (12.8) | < 0.001 | 2311 |
| admission Glucose (mg/dl) (median, IQR) | 175.5 (140 - 235) | 131.0 (114 - 155) | 119.0 (105 - 141) | < 0.001 | 2311 |
| HbA1c (%) (median, IQR) | 6.6 (6.1 - 7.4) | 5.9 (5.8 - 6.1) | 5.4 (5.2 - 5.5) | < 0.001 | 2311 |
| HbA1c (mmol/mol) (median, IQR) | 48.6 (43.2-57.4) | 41.0 (40.0-43.2) | 35.5 (33.3-36.6) | < 0.001 | 2311 |
| SHR (median, IQR) | 1.2 1.0- 1.5) | 1.1(0.9 - 1.3) | 1.1 (1.0 - 1.3) | < 0.001 | 2311 |


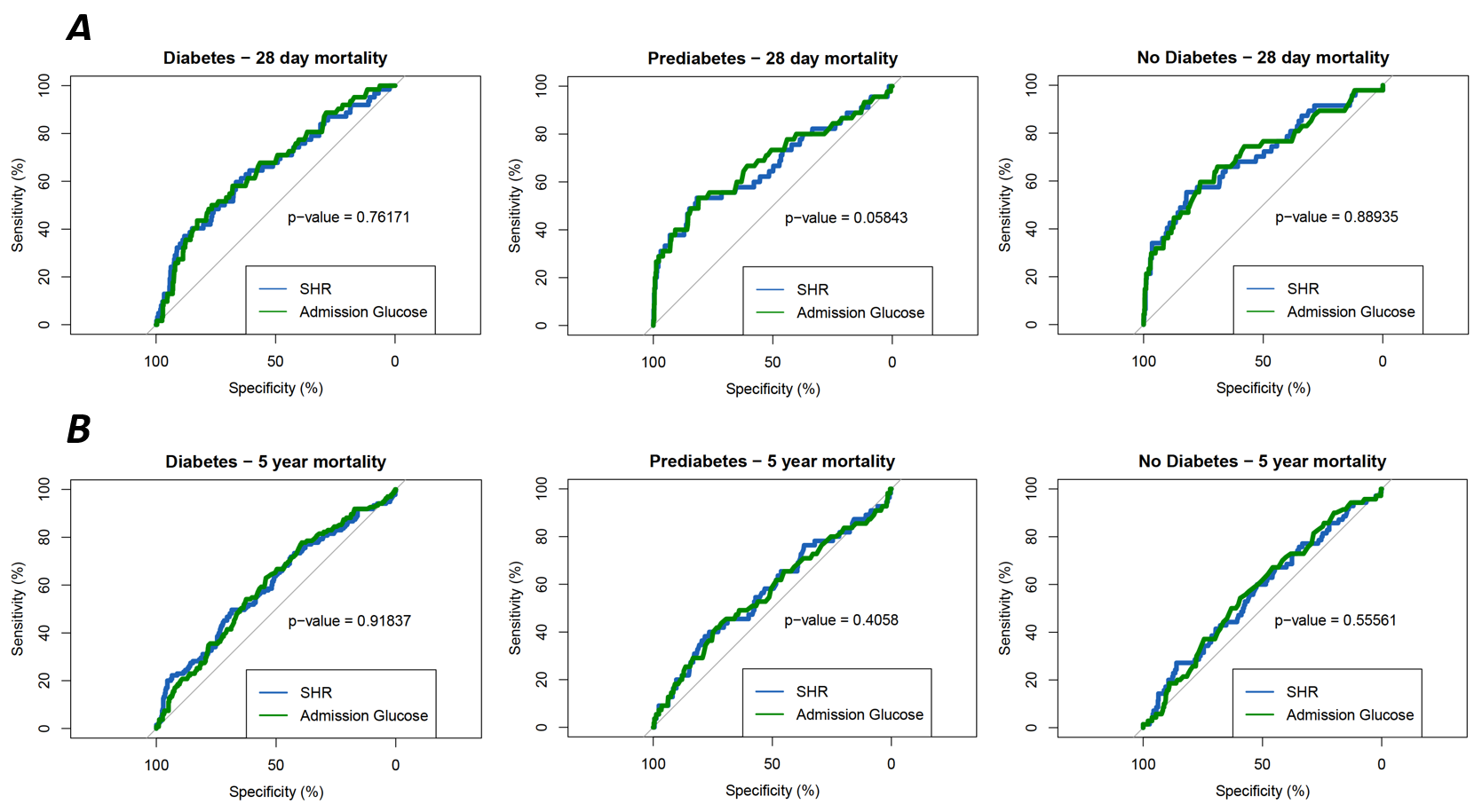


***Figure 1:*** ***A:*** *ROC curves for prediction of 28 day mortality stratified by three diabetes groups.* ***B****: ROC curves for prediction of 5-year mortality stratified by three diabetes groups. P-values for the comparison of SHR and admission glucose are calculated by using bootstrapping.*
